# Deep Reinforcement Learning-Based Trading Strategy for Load Aggregators on Price-Responsive Demand

**DOI:** 10.1155/2022/6884956

**Published:** 2022-09-12

**Authors:** Guang Yang, Songhuai Du, Qingling Duan, Juan Su

**Affiliations:** College of Information and Electrical Engineering, China Agricultural University, Beijing, China

## Abstract

With the development of the Internet of things and smart grid technologies, modern electricity markets seamlessly connect demand response to the spot market through price-responsive loads, in which the trading strategy of load aggregators plays a crucial role in profit capture. In this study, we propose a deep reinforcement learning-based strategy for purchasing and selling electricity based on real-time electricity prices and real-time demand data in the spot market, which maximizes the revenue of load aggregators. The deep deterministic policy gradient (DDPG) is applied through a bidirectional long- and short-term memory (BiLSTM) network to extract the market state features that are used to make trading decisions. The effectiveness of the method is validated using datasets from the New England electricity market and Australian electricity market by introducing a bidirectional LSTM structure into the actor-critic network structure to learn hidden states in partially observable Markov states through memory inference. Comparative experiments of the method show that the method can provide greater yield results.

## 1. Introduction

The basic feature of the electricity market is that prices follow demand and price changes affect the quantity demanded [1]. The economic operation of the electricity market will help to reduce the cost of electricity use and is an effective way of enhancing the security of the electricity system through economy [2]. The study of response characteristics in terms of timing, trading rules, etc., can enhance the flexibility of electricity markets to improve the accuracy of forecasting and decision-making [3–5]. In recent years, with the development of the Internet of things and smart grid technologies, especially the technological advancement of ambient intelligence, the widespread deployment of smart meters has equipped more customers with two-way communication capability, making price-responsive load possible. Price-responsive demand (PRD), which unifies the original price-based and incentive-based demand-side response, makes the originally uncontrollable price-based demand response a controllable resource and unifies the incentive-based demand response to the response to price.

For system operators, PRD is a reliable real-time resource that can be described as the price-adjusted load, providing a new means and tool for dispatch; for consumers, PRD reduces electricity bills and improves energy use efficiency. The EcoGrid EU trial results show that residential load could be considered price-sensitive on certain test days [6]. Moreover, the California ISO PJM and Alstom Grid results show that PRD helped improve the efficiency of market operations and significantly increase system reliability [7]. Price-responsive mechanisms facilitate the integration of new flexible energy sources and reduce rail operating costs [8].

Load aggregators can consolidate demand response customer resources and become price-responsive loads as a single large customer. To a certain extent, this eliminates uncertainty in user response behavior and allows small and medium loads to participate in the electricity market in conjunction with their own load control characteristics; aggregated demand response resources can be flexibly managed to improve response efficiency based on forecast or current electricity spot market prices. In Liu et al. [9], a hybrid stochastic/robust optimisation approach with a model that minimizes the expected net cost was proposed for distributed generation (DG), storage, dispatchable DG, and price-sensitive load bidding strategies in the pre-electricity day market. The results show that the wind power output had a negative correlation with the price-based demand response load response, and the correlation could reduce the system operating cost and improve the economy of system dispatch. In Geng et al. [10], a two-stage stochastic market power purchase model with DR resources was constructed to minimize the energy purchase cost of integrated energy service providers in different types of markets, and the impact of flexible heating load on their power purchase strategy was presented. In the previous day's market, a multi-time scale stochastic optimal scheduling model for electric vehicle (EV) charging stations with demand response was proposed with the objective of minimizing the daily operating cost and introducing price-based demand response to optimise the net load curve of charging stations [11]. Combining the price-based demand response measures, the optimisation was proposed with the objectives of maximizing the revenue of EV load aggregators and minimizing the load fluctuation [12].

Heuristic algorithms, meta-heuristics, and intelligent evolutionary algorithms for the optimal solution of decision problems are used in various fields. In Zhao and Zhang [13], a learning-based generalisation algorithm is proposed to improve generalisation by adjusting the evolutionary strategy of the algorithm based on feedback information in the optimisation process according to the actual problem. Pasha et al. [14] present an integrated optimisation model whose objective is to maximize the total turnover profit generated by the transport business and solve the proposed model through a decomposition-based heuristic algorithm. Kavoosia et al. [15] propose an evolutionary algorithm to solve the developed mathematical model, implemented through an enhanced adaptive parameter control strategy that effectively varies the algorithm parameters throughout the search process. Dulebenets' [16] study proposes a new adaptive polymorphic memory algorithm to solve the scheduling problem of transport and to help operators in proper operational planning. Rabbani et al.'s [17] study presents a mixed integer linear programming model to find the optimal route sequence and minimize time consumption through non-dominated sequential genetic algorithm II and multi-objective particle swarm optimisation.

In recent years, deep reinforcement learning has autonomous recognition and decision-making capabilities and has been successfully applied in the energy sector [18–20]. The feasibility of using it for grid regulation has also been demonstrated [21, 22]. The requirements associated with demand response can be met [23]. Reinforcement learning theory represents a mathematical model of learning that is rewarded by repeated trial and error and is based on the psychological term operant conditioning, which derives its name from the phenomenon of the increased frequency of autonomous behavior reinforcement. A customer agent model was proposed in [24] applying reinforcement learning Q-learning for predicting price-sensitive load reductions. A pricing strategy was investigated in [25] for charging station operators based on noncooperative games and deep reinforcement learning, and the effectiveness of the proposed framework was validated with real data from cities. Moreover, a real-time pricing technique was proposed in [26] based on multi-intelligent reinforcement learning, and it worked well in producing consumer-driven applications of mini-smart grids. The researchers behind [27] considered thermostatically controlled loads, energy storage systems (ESS), and price-responsive loads for flexible demand-side dispatch of microgrids based on deep reinforcement learning, which significantly reduced input costs. The researchers of [28] gave a dynamic pricing strategy based on DDPG considering the historical behavior data of electric vehicles, peak-valley time-sharing tariff, and the demand-side response pattern to guide the customer tariff behavior and exploit the economic potential of the electricity market. Considering the cooperation between wind farms and electric vehicles, an intelligent pricing decision was proposed in [29] for EV load aggregators based on deep reinforcement learning algorithms to achieve an increase in overall economic benefits. To maximize the long-term revenue of electricity sellers under the electricity spot market, the researchers of [30] proposed a dynamic optimisation scheme for demand response using reinforcement learning. For the price difference between the day-ahead and real-time markets in the electricity spot market, the researchers of [31] achieved an effective solution for the optimal bidding strategy based on deep reinforcement learning. Further, an improved deep deterministic policy gradient algorithm was proposed in [32] as a building-level control strategy to improve the distributed electric heating load-side demand response capability. A dual DQN agent was proposed in [33] to evaluate the elasticity of power systems. Other research [34] combined the cross-entropy method (CEM), the maximum mean difference method (MMD), and the deep deterministic policy gradient algorithm with twin delays (TD3) in evolutionary strategies to propose the diversity evolutionary strategy deep reinforcement learning (DEPRL).

In summary, load aggregators, acting on behalf of small and medium electricity consumers in price-responsive load trading, face the problem of how to purchase electricity from the market and sell it to consumers and need to optimise their decision-making options in terms of both purchases and sales in order to maximize profits. Therefore, it is necessary to study the buying and selling strategies of price-responsive loads that can be carried out by load aggregators in dynamic trading in the electricity spot market. It is also necessary to overcome the problem of the slow training convergence rate when the input dimension of reinforcement learning is too large. Based on the above problems, this study proposes a deep reinforcement learning method based on BiLSTM for load aggregators to purchase and sell electricity, taking the maximum revenue of load aggregators under the price-responsive load mechanism as the scenario. The contributions of this study are as follows.

We propose a BiLSTM-DDPG model to make the trading strategy for load aggregators. We describe the trading process as a partially observable Markov decision process (POMDP). The bidirectional LSTM neural network is used to process the bidirectional time axis state information one by one and generate bidirectional coded information to cope with the dynamic changes in an uncertain environment. We propose the BiLSTM-DDPG method, which integrates time-domain processing and has autonomous recognition and decision-making capabilities. BiLSTM can extract features and temporal relationships, avoiding gradient disappearance and gradient explosion. DDPG allows for more accurate recognition and optimal decision-making for complex electricity spot market environments.

## 2. Materials and Methods

### 2.1. BiLSTM Model

**Figure 1:**
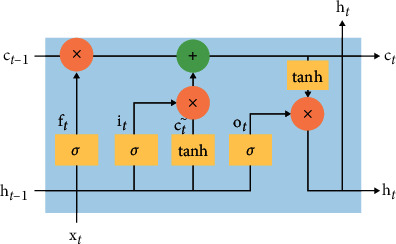
Neuron structure of LSTM.

The recurrent neural network (RNN) is a neural network that processes temporal data as input to itself. In a single computational unit, the data (*x*_*t*_) from the previous *t* moments and the computational output (*h*_t_ _−_ _1_) from the previous *t* − 1 moments are used as input, and in the unit output, in addition to the output *y*_*t*_, ht is also generated, and the data are passed on to the next moment (*t* + 1) for the next computation. The RNN based on this design structure has predictive capability. LSTM is an improved RNN, and compared with RNN, LSTM adds the forgetting gate at the output and implements the forgetting function by a state parameter (c). The LSTM structure is shown in Figure 1.

The LSTM cell contains an oblivion gate, an input gate, and an output gate. The oblivion gate (ft) selectively forgets the information of the previous cell, as shown in equation (1); it takes the information of the previous cell and the current state as input and outputs a value from 0 to 1 by the sigmoid function, and this value is the percentage of retained transmission information. The current cell input information proportion is controlled by the input gate, as shown in (2), *C*^*∼*^ is the proportion of retained information, as shown in (3), and (4), representing *C*_*t*_, weights the retained information and new information as the current cell state. The output gate determines how much information is output, and (5) and (6) pass some of the information from the current cell to the later cells [35–37]. The DDPG algorithm with LSTM added stores and passes on information about the trend of the hidden state of the environment in the time domain, as shown in the following equations:

(1) $$\begin{array}{c} f_{t}=\mathrm{sigmoid}\left(W_{f}\cdot \left[h_{t-1},x_{t}\right]+b_{f}\right), \end{array}$$

(2) $$\begin{array}{c} i_{t}=\mathrm{sigmoid}\left(W_{i}\cdot \left[h_{t-1},x_{t}\right]+b_{i}\right), \end{array}$$

(3) $$\begin{array}{c} C^{∼}=\tanh\left(W_{c}\cdot \left[h_{t-1},x_{t}\right]+b_{c}\right), \end{array}$$

(4) $$\begin{array}{c} C_{t}=f_{t-1}⊙C_{t-1}+i_{t}⊙C, \end{array}$$

(5) $$\begin{array}{c} o_{t}=\mathrm{sigmoid}\left(W_{o}\cdot \left[h_{t-1},x_{t}\right]+b_{o}\right), \end{array}$$

(6) $$\begin{array}{c} h_{t}=o_{t}\tanh\left(C_{t}\right). \end{array}$$

**Figure 2:**
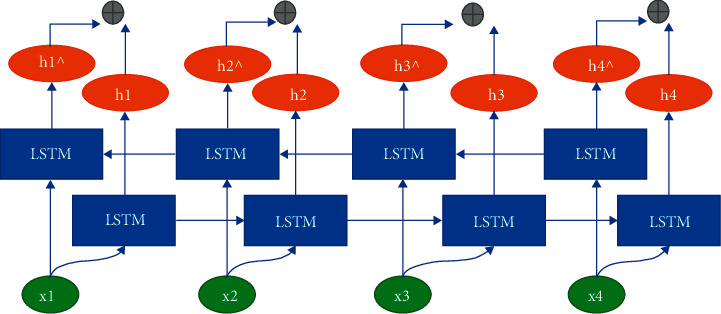
Neuron structure of BiLSTM.

The BiLSTM propagates the state of the hidden layer using a timeline of “from the past to the future” and “never to the past” directions, as shown in Figure 2. The BiLSTM captures the transformation pattern of features on a bidirectional time axis. In the figure, LSTM1 and LSTM2 are the forward and reverse LSTM models, respectively. The output at moment *h*_t_ can be expressed as follows:

(7) $$\begin{array}{c} h_{t}=LSTM1\left(H_{t-1},H_{t}\right)+LSTM2. \end{array}$$

### 2.2. Reinforcement Learning

The mathematical basis for reinforcement learning is the Markov decision process (MDP), which consists of a state space, an action space, a state transfer matrix, a reward function, and a discount factor. The MDP tries to find a strategy that allows the system to obtain the maximum cumulative reward value. The state is a generalisation of the current environment; the state space is the set of all possible states, denoted as S; action is the decision made; the action space is the set of all possible actions, denoted as A; the agent is the subject doing the action; and the policy function is the decision to control the action of an intelligent body based on the observed state.

**Figure 3:**
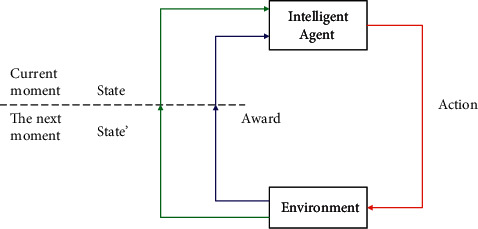
An agent interacts with the environment.

Agent environment interaction (AEI) is when an intelligent body observes the state of the environment (s) and makes an action (a), the action changes the state of the environment, and the environment gives the intelligent body a reward (*r*) and a new state (s′), as shown in Figure 3.

In this study, MDPs can be expressed as *(S, O, A, P, r, γ, S*), where S is a set of consecutive states, and *A* is a series of consecutive actions. *P:S×A×S⟶R* is the transfer probability function, *r:S×A⟶R* is the reward function, *γ* is the discount factor, *S* is the initial state distribution, and *O* is the set of continuous partial observations corresponding to the states in *S*. In training, *S*_0_ is obtained by sampling from the initial state distribution S. At each time step *t*, the intelligence determines the current ambient state space (*S*_t_ ∈ *S*). The reward *r*: *S* × *A* ⟶ *R* is obtained by taking the action *a*_*t*_∈*π* (*s*_*t*_) according to the strategy *π: S ⟶ A*, and the new ambient state *S*_*t*_+1 is obtained.

The goal of the intelligent body is to maximize the expected return, as follows:

(8) $$\begin{array}{c} Es\left[R_{0}|S\right]. \end{array}$$

The payoff is the discounted sum of future returns, as follows:

(9) $$\begin{array}{c} R_{t}=\sum_{i=t}^{\infty }\gamma^{i-t}r_{i}. \end{array}$$

The *Q* function is defined as follows:

(10) $$\begin{array}{c} Q^{\pi }\left(s_{t},a_{t}\right)=E\left[R_{t}|s_{t},a_{t}\right]. \end{array}$$

In the partially observable case, an agent acts on partial observations, *a*_t_ = *π(O*_*t*_), where *O*_t_ is the partial observation corresponding to the complete state (*S*_t_).

### 2.3. DDPG Model

**Figure 4:**
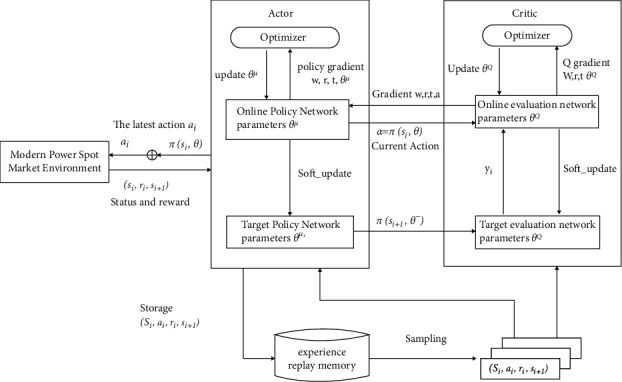
Network structure of DDPG.

The DDPG algorithm incorporates the ideas of DQN and uses a deterministic policy function to enable the problem to perform better on continuous spaces of high dimensionality. The learning framework for deterministic strategies takes the approach of the actor-critic algorithm, where the actor is the action strategy, and the critic is the evaluation, which in this case estimates the value function using function approximation methods. The network structure of DDPG is shown in Figure 4.

DDPG uses two neural networks to represent the deterministic strategy *A* = *π*_*θ*_(s) and the value function Q^*μ*^(s, A). The network parameters are *θ* and *μ*, where the strategy neural network is used to update the behavioral strategy of the intelligence, corresponding to the actor network in the actor-critic structure, and the value network is used to approximate the value function and provide gradient information for the update of the strategy network, corresponding to the critic network in the actor-critic structure. DDPG finds an optimal strategy *π*_*θ*_ to maximize the expected return, as follows:

(11) $$\begin{array}{c} J\left(\theta \right)=E_{si∼p\pi ,ai∼\pi }\left[R_{0}\right]. \end{array}$$

A parameter update of policy network by the gradient ∇_*θ*_*J*(*θ*) is as follows:

(12) $$\begin{array}{c} \nabla_{\theta }J\left(\theta \right)=E_{\left(s∼p\pi \right)}\left[{\nabla_{a}Q^{\pi }\left(s,a\right)|}_{a=\pi \left(s\right)}\nabla_{\theta }\pi_{\theta }\left(s\right)\right]. \end{array}$$

The expected return value after taking action A in state S, following strategy *π*, is as follows:

(13) $$\begin{array}{c} Q^{\pi }\left(s,a\right)|=E_{s_{i}∼p_{\pi },a_{i}∼\pi }\left[R_{t}|s,a\right]. \end{array}$$

The value network is updated according to the value-network-updating method in DQN; namely, the loss minimization function *L(μ)* is used to update the value network parameters, as shown in the following equations:

(14) $$\begin{array}{c} L\left(\mu \right)=E_{s_{t},a_{t},r\left(s_{t},a_{t}\right),s_{t+1}}\left[{\left(y_{t}-Q_{t}\left(s_{t},a_{t}\right)\right)}^{2}\right], \end{array}$$

(15) $$\begin{array}{c} y_{t}=r\left(s_{t},a_{t}\right)+\gamma Q_{\mu }\left(s_{t+1},a_{t+1}\right), \end{array}$$

(16) $$\begin{array}{c} a_{t+1}∼\pi_{\theta }\left(s_{t+1}\right), \end{array}$$

where *θ*′' and *μ*′' denote the target actor network and target critic network parameters, respectively. DDPG uses a data playback mechanism to obtain training samples [38–41]. The information about the gradient of the Q-value function regarding the action of the intelligent body is passed to the actor network through the critic network, and the update of the policy network is performed in the direction of boosting the Q-value according to (16).

### 2.4. BiLSTM-DDPG-Based Trading Strategy for Load Aggregators on PRD

**Figure 5:**
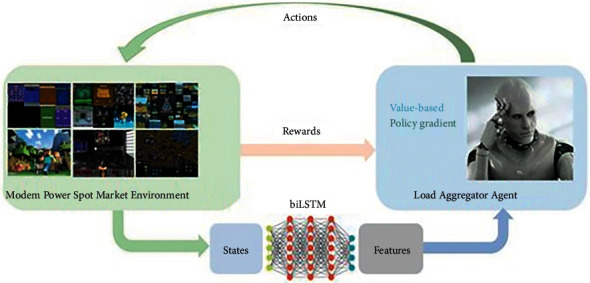
BiLSTM-DDPG model processing steps for power markets.

**Figure 6:**
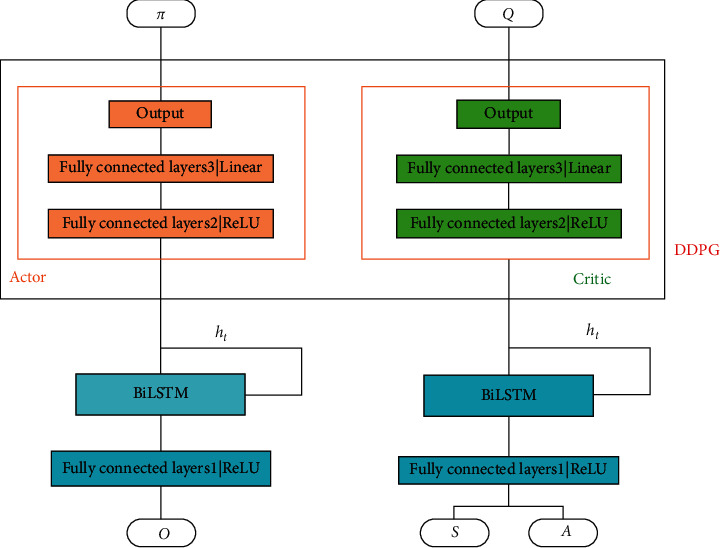
Structure of the actor-critic network.

The description of the variables of the BiLSTM-DDPG-based trading strategy for load aggregators on PRD is shown in Table 1. The BiLSTM-DDPG model processing steps for power markets are shown in Figure 5. The DDPG deep reinforcement learning with BiLSTM structure is based on the actor-critic network structure, shown in Figure 6.

For load aggregators, the main objective of participating in price demand response is to maximize the benefits of energy trading. The total benefits received by the load aggregator in the real-time market are as follows:

(17) $$\begin{array}{c} \max R=R^{RT}-C^{RT}-C^{DA}, \end{array}$$

where *R*^RT^ is the profit on electricity sales in the real-time market, *C*^RT^ is the cost of electricity purchased by the electricity seller in the real-time market, and *C*^DA^ is the cost of electricity purchased in the day-ahead market.

(18) $$\begin{array}{c} \max R=R^{RT}=\sum_{t\in T}\lambda_{t}^{RT}-P_{t,s}^{RT}-\sum_{t=1}^{T}\lambda_{t}^{RT}+P_{t,p}^{RT}-\sum_{t=1}^{T}\lambda_{t}^{DA}P_{t}^{DA}, \end{array}$$

where *λ*_*t*_^*RT*−^ is the selling price in the real-time market at time *t*, and *P*_*t*,*s*_^*RT*^ is the amount of electricity sales. *λ*_*t*_^*RT*+^ is the purchase price in the real-time market at time *t*, and *P*_*t*,*p*_^*RT*^ is the amount of electricity purchased. *λ*_*t*_^*DA*^ is the purchase price in the day-ahead market at time *t*, and *P*_*t*_^*DA*^ is the amount of electricity purchased.

The input of the neural network is the state, and the output is action value. The neural network consists of three full connection layers; the first two layers are activated by the rectified linear unit function, and the third layer is the linear connection. The agent is built according to the logic of the pseudo-code, obtaining the reward values, iterating through the Bellman equation, and then gradient descending the difference between the target network and the action network, where the target network is updated using the soft update method. The parameters and description of DDPG algorithm used in the case are shown in Table 2.

The DDPG that introduces the BiLSTM needs to use the before-and-after order of states during training, so the corresponding experience pool data are saved as a sequence of whole sets to provide experience data for subsequent updates of the actor and critic networks, and the sequence of saved data is as follows:

(19) $$\begin{array}{c} o_{1},a_{1},r_{1},s_{1},o_{2},a_{2},r_{2},s_{2}...o_{T},a_{T},r_{T},s_{T}, \end{array}$$

where *T* is the number of steps per set. When the number of time steps is a multiple of *T*, the program structure is cleared of historical data records, and the empirical data are recoded. We can reconstruct observable historical information and full-state historical information from empirical data, as follows:

(20) $$\begin{array}{c} c_{t}=\left(s_{1},a_{1},...,a_{t-1},s_{t}\right),\\ h_{t}=\left(o_{1},a_{1},...,a_{t-1},o_{t}\right). \end{array}$$

The critic and actor networks are updated separately. As BiLSTM is a time-series-based RNN, the updates to the critic and actor networks are backpropagated through time (BPTT), and the updates are as follows:

(21) $$\begin{array}{c} \Delta \mu =\frac{1}{NT}\sum \sum \left(y_{t}^{i}-Q^{\mu }\left(c_{t}^{i},a_{t}^{i}|\mu \right)\right)\frac{\partial Q^{\mu }\left(h_{t}^{i},a_{t}^{i}\right)}{\partial \mu },\\ \Delta \theta =\frac{1}{NT}\sum \sum \frac{\partial Q^{\mu }\left(c_{t}^{i},\pi^{\theta }\left(h_{t}^{i}\right)\right)}{\partial a}\frac{\partial \pi^{\theta }\left(h_{t}^{i}\right)}{\partial \theta }. \end{array}$$

The pseudo-code of BiLSTM-DDPG is as follows:

  The parameters *μ* and *θ* initialize the critic network *Q*_*μ*_(*a*_*t*_, *s*_*t*_) and the actor network *π*^*θ*^(*h*^t^), respectively;
 
*μ′*← *μ*,*θ′*←*θ* initialize the target networks Q^*μ*^ and *π*^*θ*^;
  Initialize the experience replay area ®:
  for episode = 1,. .., *M* do
  Clear the history information *h*_*0*_ and *c*_*0*_;
  For *t* = 1,. .., *T* do
  Get observation (*o*_*t*_) and full state (*S*_*t*_.) from environment
  Update the history information, *h*_*t*_ ← *h*_*t*−1_, *a*_*t*−1_, *o*_*t*_;
  Generate action, *a*_*t*_=*π*^*θ*^(*h*_*t*_)+*ξ*;
  End for
  Store the empirical sequence (*o*_1_, *a*_1_, *r*_1_, *s*_1_, *o*_2_, *a*_2_, *r*_2_, *s*_2_...*o*_*T*_, *a*_*T*_, *r*_*T*_, *s*_*T*_) into the experience pool *R*;
  Sample N episodes of experiences in experience pool *R*(*o*_1_^*i*^, *a*_1_^*i*^, *r*_1_^*i*^, *s*_1_^*i*^, *o*_2_^*i*^, *a*_2_^*i*^, *r*_2_^*i*^, *s*_2_^*i*^...*o*_*T*_^*i*^, *a*_*T*_^*i*^, *r*_*T*_^*i*^, *s*_*T*_^*i*^)_*i*=1,...,*N*_;
  Construct partial observable history, *h*_*t*_^*i*^=(*o*_1_^*i*^, *a*_1_^*i*^, ..., *a*_*t*−1_^*i*^, *o*_*t*_^*i*^);
  Construct the full state history message, *c*_*t*_^*i*^=(*s*_1_^*i*^, *a*_1_^*i*^, ..., *a*_*t*−1_^*i*^, *s*_*t*_^*i*^);
  Calculate the target value of each sample, (*y*_1_^*i*^, ...*y*_*T*_^*i*^);
  Update the critic network: Δ*μ*=1/*NT*∑∑(*y*_*t*_^*i*^ − *Q*^*μ*^(*c*_*t*_^*i*^, *a*_*t*_^*i*^*|μ*))*∂Q*^*μ*^(*h*_*t*_^*i*^, *a*_*t*_^*i*^)/*∂μ*;
  Update the actor network: Δ*θ*=1/*NT*∑∑*∂Q*^*μ*^(*c*_*t*_^*i*^, *π*^*θ*^(*h*_*t*_^*i*^))/*∂a∂π*^*θ*^(*h*_*t*_^*i*^)/*∂θ*;
  Update target network: *μ*′←*τμ*+(1 − *τ*)*μ*′; *θ*′←*τθ*+(1 − *τ*)*θ*′;
  End for

## 3. Results and Discussion

### 3.1. Experimental Settings

The experimental environment is as follows: Python 3.6.2, TensorFlow 2.0.0a GPU, Intel(R) Core(TM) i5-7200U@2.50 GHz∼2.70 GHz, 64 bit, 8 GB of RAM, and NVIDIA GeForce 940MX.

The first dataset is the annual whole-point data of the New England electricity market (ISO-NE) in the United States, selected for the Connecticut Region [42]. Real-time electricity price data are collected for 1,917 consecutive days from January 1, 2016, to March 31, 2022, at a frequency of once per hour, for a total of 46,008 moments.

The second dataset is the annual whole-point data of the Australian Energy Market Operator (AEMO) in the Australian, selected for the Connecticut New South Wales Region [43]. Real-time electricity price data are collected for 1186 consecutive days from January 1, 2018, to September 30, 2021, at a frequency of once per half hour, for a total of 56,928 moments.

### 3.2. Experiment 1: Hourly Load Aggregator Trading Strategy in ISO-NE

In this experiment, the prediction of three days' worth of trading strategy from January 1, 2016, to March 28, 2022, is used as the training set, data from March 29, 2022, to March 30, 2022, as the validation set, and data from April 1, 2021, to April 3, 2021, as the test set.

**Figure 7:**
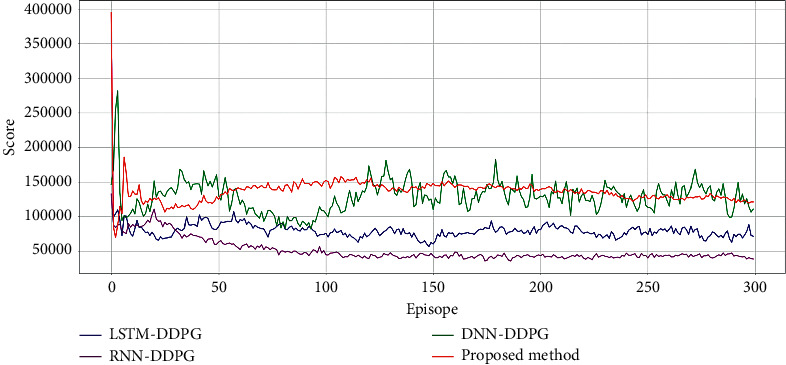
Profit curves of DNN-DDPG, RNN-DDPG, LSTM-DDPG, and the proposed method in ISO-NE.

**Figure 8:**
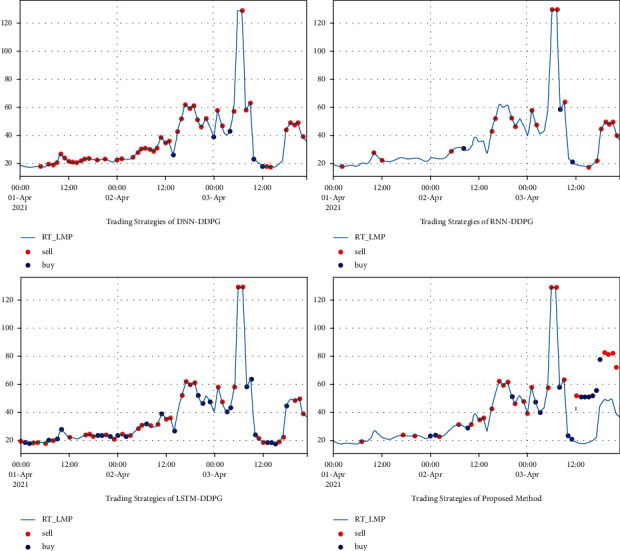
Trading strategies of DNN-DDPG, RNN-DDPG, LSTM-DDPG, and the proposed method from April 1, 2021, 0:00 pm, to April 2, 2021, 4:30 am, in ISO-NE.

The comparison of the profit curves for the hourly load aggregator trading strategy in ISO-NE is shown in Figure 7; the performance of buying and selling is shown in Table 3; trading strategies from April 1, 2021, 0:00, to April 2, 2021, 4:30, in the ISO-NE results are shown in Figure 8. The overall evaluation from April 1, 2021, 0:00, to April 2, 2021, 4:30, in ISO-NE is shown in Table 4. It is demonstrated that the proposed method is more economical than DNN-DDPG, RNN-DDPG, and LSTM-DDPG, indicating ith as better convergence ability.

### 3.3. Experiment 2: Load Aggregator's Trading Strategy Every Half Hour for 2 Days in AEMO

In this experiment, the prediction of every half hour for two days' trading strategy from January 1, 2018, to September 27, 2021, is used as the training set, data from September 28, 2021, to September 29, 2021, are used as the validation set, and data from September 30, 2021, are used as the test set.

**Figure 9:**
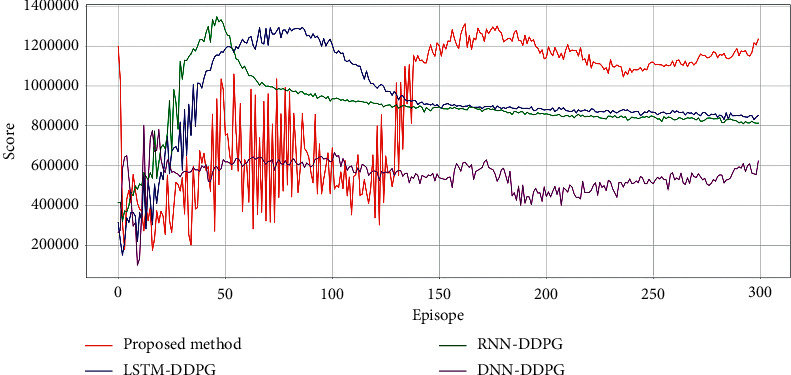
Profit curves of DNN-DDPG, RNN-DDPG, LSTM-DDPG, and the proposed method in AEMO.

**Figure 10:**
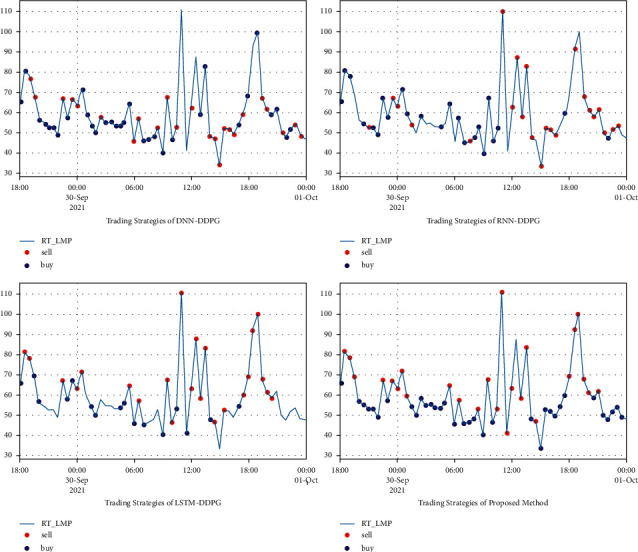
Trading strategies of DNN-DDPG, RNN-DDPG, LSTM-DDPG, and BiLSTM-DDPG from September 29, 2021, 18:00, to October 1, 2021, 0:00, in AEMO.

The comparison of the profit curves for the hourly load aggregator trading strategy in AEMO is shown in Figure 9; the performance of buying and selling is shown in Table 5. Trading strategies from April1, 2021, 0:00, to April 2, 2021, 4:30, in AEMO results are shown in Figure 10. The overall evaluation from April 1, 2021, 0:00, to April 2, 2021, 4:30, in AEMO is shown in Table 6. It is also demonstrated that the proposed method is more economical than for DNN-DDPG, RNN-DDPG, and LSTM-DDPG, indicating ith as better convergence ability.

## 4. Conclusions

This study investigates deep reinforcement learning in load aggregators' participation in the electricity spot real-time market trading strategy. The proposed improved DDPG algorithm can be used for load aggregators' real-time load purchase and sale transactions in the electricity spot real-time market. The main work is as follows: (1) an improved BiLSTM-DDPG with better convergence ability is proposed to solve the problem that DDPG does not easily converge when the input dimension is too large; (2) deep reinforcement learning is introduced into the analysis of power purchase and sale strategies in the electricity spot market so that load aggregators can participate in demand response with better results; and (3) in the case of IOS-NE and AEMO, it is proved that under the strategy implemented by the proposed method, it is more economical for the load aggregator to participate in the price-responsive load than for DNN-DDPG, RNN-DDPG, and LSTM-DDPG.

The proposed algorithm can be used to solve scenarios with large data volumes and high requirements for timeliness in the electricity market, providing an idea for the study of optimisation problems. This study focuses on the load aggregator's purchase and sale model, but has not studied the point-to-point user. Future research will combine transfer learning and federal learning to achieve distributed peer-to-peer transaction optimisation in electricity retail market.

## Figures and Tables

**Table 1:** Description of the variables of the DDPG.

Name	Meaning	Meaning of this study
Agent	Intelligence to be controlled	Price load-responsive load aggregator
State, S	Status of the agent	Current electricity spot market price (*λ*_*t*_^*RT*^, *λ*_*t*_^*DA*^)
Action, a	Actions that an agent can take	Purchase and sale of electricity (output *sell/buy*)
Reward, r	Timely return value of the environment used to evaluate the quality of an action on an agent	Revenue from load aggregators *λ*_*t*_^*RT*−^*P*_*t*,*s*_^*RT*^ − *λ*_*t*_^*RT*+^*P*_*t*,*p*_^*RT*^ − *λ*_*t*_^*DA*^*P*_*t*_^*DA*^
Policy, P	Agent decides the strategy of the next action based on the current state	Buying and selling actions in the next cycle are determined based on the status of the load aggregator in the previous cycle
Value	Return value of an agent action's long-term value, distinguished from the short-term return represented by reward	Total revenue over the period of operation ∑_*t*∈*T*_*λ*_*t*_^*RT*^ − *P*_*t*,*s*_^*RT*^ − ∑_*t*=1_^*T*^*λ*_*t*_^*RT*^+*P*_*t*,*p*_^*RT*^ − ∑_*t*=1_^*T*^*λ*_*t*_^*DA*^*P*_*t*_^*DA*^
Environment	Environment of the agent	Fluctuations in electricity prices (input *t* real-time locational marginal price, input *t* real-time *l* demand)

**Table 2:** Description of the parameters of the DDPG.

Parameters	Meaning	Value
TAU	Smoothing coefficient of target network in actor and critic network	0.001
*α*	Actor network and critic network learning rate	0.0005
Batch_size	Number drawn from the experience pool per training	64
Capacity	Size of the experience pool	100000
*γ*	Discount factor	0.99

**Table 3:** Performance of buying and selling of DNN-DDPG, RNN-DDPG, LSTM-DDPG, and proposed method.

Time	Real-time demand	Real-time LMP	DNN-DDPG strategy	RNN-DDPG profit	LSTM-DDPG profit	Proposed method profit
2021/4/1 0:00	2180.29	18.58	—	—	Sell	—
2021/4/1 1:00	2128.78	17.66	—	—	Buy	—
2021/4/1 2:00	2129.59	16.97	—	Sell	Buy	—
2021/4/1 3:00	2199.59	17.39	—	—	Sell	——
2021/4/1 4:00	2396.96	17.47	—	—	Sell	—
2021/4/1 5:00	2726.9	17.76	Sell	—		—
2021/4/1 6:00	3025.03	17.19	—	—	Sell	—
2021/4/1 7:00	3184.98	19.24	Sell	—	Buy	Sell
2021/4/1 8:00	3232.86	19	Sell	—	Sell	—
2021/4/1 9:00	3189.72	20.15	Sell	—	Buy	—
2021/4/1 10:00	3158.38	26.81	Sell	Sell	Buy	—
2021/4/1 11:00	3124.1	23.9	Sell	—		—
2021/4/1 12:00	3140.79	21.25	Sell	Sell	Sell	—
2021/4/1 13:00	3124.51	20.76	Sell	—		—
2021/4/1 14:00	3095.21	20.12	Sell	—		—
2021/4/1 15:00	3099.09	21.59	Sell	—		—
2021/4/1 16:00	3160.18	22.99	Sell	—	Sell	—
2021/4/1 17:00	3215.54	23.36	Sell	—	Sell	Sell
2021/4/1 18:00	3318.29	22.24	—	—	Sell	—
2021/4/1 19:00	3275	22.64	Sell	—	Buy	—
2021/4/1 20:00	3149.49	22.73	—	—	Buy	Sell
2021/4/1 21:00	2920.19	23.02	Sell	—	Sell	—
2021/4/1 22:00	2696.89	21.73	—	—	Buy	—
2021/4/1 23:00	2536.62	20.57	—	—	Sell	—
2021/4/2 0:00	2456.54	22.66	Sell	—	Buy	Buy
2021/4/2 1:00	2422.44	23.39	Sell	—	Sell	Buy
2021/4/2 2:00	2429.74	22.41	—	—	Buy	Sell
2021/4/2 3:00	2496.1	22.73	—	—	Sell	—
2021/4/2 4:00	2665.92	24.23	Sell	—		—
2021/4/2 5:00	2909.75	27.85	Sell	Sell	Sell	—
2021/4/2 6:00	3073.85	29.92	Sell	—	Sell	—
2021/4/2 7:00	3107.32	30.83	Sell	—	Buy	Sell
2021/4/2 8:00	3160.12	30.14	Sell	Buy	Sell	—
2021/4/2 9:00	3185.16	28.23	Sell	—		Buy
2021/4/2 10:00	3207.49	30.96	Sell	—	Sell	Sell
2021/4/2 11:00	3191.83	38.49	Sell	—	Buy	—
2021/4/2 12:00	3172.89	34.4	Sell	—	Sell	Sell
2021/4/2 13:00	3132.9	35.41	Sell	—	Sell	Sell
2021/4/2 14:00	3168.14	25.88	Buy	—	Buy	—
2021/4/2 15:00	3249.82	42.4	Sell	Sell		Sell
2021/4/2 16:00	3350.11	51.55	Sell	Sell	Sell	—
2021/4/2 17:00	3368.91	61.76	Sell	—	Sell	Sell
2021/4/2 18:00	3435.6	59.28	Sell	—	Sell	Sell
2021/4/2 19:00	3389.91	60.96	Sell	—	Sell	Sell
2021/4/2 20:00	3286.39	51.38	Sell	Sell	Buy	Buy
2021/4/2 21:00	3081.24	45.9	Sell	Sell	Buy	Sell
2021/4/2 22:00	2865.53	51.58	Sell	—		—
2021/4/2 23:00	2706.24	46.81	—	—	Buy	Sell
2021/4/3 0:00	2611.68	38.81	Buy	—		Sell
2021/4/3 1:00	2569.03	57.57	Sell	Sell	Sell	Sell
2021/4/3 2:00	2564.6	46.78	Sell	Sell	Sell	Buy
2021/4/3 3:00	2595.77	39.73	—	—	Buy	Buy
2021/4/3 4:00	2693.64	42.5	Buy	—	Buy	—
2021/4/3 5:00	2831.29	57.37	Sell	—	Sell	Sell
2021/4/3 6:00	2929.99	129.03	—	Sell	Sell	Sell
2021/4/3 7:00	2928.95	128.95	Sell	Sell	Sell	Sell
2021/4/3 8:00	2857.46	57.82	Sell	Buy	Buy	Buy
2021/4/3 9:00	2759.64	62.91	Sell	Sell	Buy	Sell
2021/4/3 10:00	2674.62	23.04	Buy	—	Buy	Buy
2021/4/3 11:00	2579.64	20.47	—	Buy	Sell	Buy
2021/4/3 12:00	2487.56	18.16	Buy	—	Sell	Sell
2021/4/3 13:00	2487.56	17.86	Sell	—	Buy	Buy
2021/4/3 14:00	2404.29	17.37	Sell	—	Buy	Buy
2021/4/3 15:00	2417.56	17.2	—	Sell	Buy	Buy
2021/4/3 16:00	2537.5	18.13	—	—	Sell	Buy
2021/4/3 17:00	2728.21	21.46	—	Sell	Sell	Buy
2021/4/3 18:00	2905.54	44.17	Sell	Sell	Buy	Buy
2021/4/3 19:00	3069.5	48.84	Sell	Sell		Sell
2021/4/3 20:00	3064.05	47.75	Sell	Sell	Sell	Sell
2021/4/3 21:00	2989.98	48.56	Sell	Sell	Sell	Sell
2021/4/3 22:00	2823.51	38.67	Sell	Sell	Sell	Sell
2021/4/3 23:00	2654.93	35.7	—	—		—
Total_Profit			87.03	14.08	189.76	215.46

**Table 4:** Overall evaluation of DNN-DDPG, RNN-DDPG, LSTM-DDPG, and the proposed method from April 1, 2021, 0:00, to April 2, 2021, 4:30, in ISO-NE.

Time	April 1, 2021, 0:00 pm, to April 2, 2021, 4:30 am
Statistical metrics	Total_profit _($/MWh)_
DNN-DDPG	44.18
RNN-DDPG	14.08
LSTM-DDPG	189.76
Proposed method	215.46

**Table 5:** Performance of buying and selling of DNN-DDPG, RNN-DDPG, LSTM-DDPG, and the proposed method from September 29, 2021, 18:00, to October 1, 2021, 0:00, in AEMO.

Time	Real-time demand	Real-time LMP	DNN-DDPG profit	RNN-DDPG strategy	LSTM-DDPG profit	Proposed method profit
2021/9/29 18:00	8870.65	65.73	Buy	Buy	Buy	Buy
2021/9/29 18:30	9037.17	81.35	Buy	Buy	Sell	Sell
2021/9/29 19:00	9039.05	78.33	Sell	Buy	Sell	Sell
2021/9/29 19:30	8765.96	68.97	Sell	—	Buy	Sell
2021/9/29 20:00	8450.8	56.96	Buy	—	Buy	Buy
2021/9/29 20:30	8257.22	55.32	Buy	Buy	—	Buy
2021/9/29 21:00	8101.09	52.93	Buy	Sell	—	Buy
2021/9/29 21:30	7898.79	53.09	Buy	Buy	—	Buy
2021/9/29 22:00	7686.03	49.27	Buy	Buy	—	Buy
2021/9/29 22:30	7684.66	67.26	Sell	Buy	Sell	Sell
2021/9/29 23:00	7561.34	57.26	Buy	Buy	Buy	Buy
2021/9/29 23:30	7582.95	66.92	Sell	Sell	Buy	Sell
2021/9/30 0:00	7541.54	63.21	Sell	Sell	Sell	Sell
2021/9/30 0:30	7430.72	71.68	Buy	Buy	Sell	Sell
2021/9/30 1:00	7324.55	59.45	Buy	Buy	—	Sell
2021/9/30 1:30	7110.78	54.4	Buy	Sell	Buy	Buy
2021/9/30 2:00	6852.24	50.24	Buy	—	Buy	Buy
2021/9/30 2:30	6631.32	58.08	Sell	Buy	—	Buy
2021/9/30 3:00	6377	54.73	Buy	—	—	Buy
2021/9/30 3:30	6211.36	55.31	Buy	—	—	Buy
2021/9/30 4:00	6079.36	53.53	Buy	—	—	Buy
2021/9/30 4:30	6104.4	53.38	Buy	Buy	Buy	Buy
2021/9/30 5:00	6187.16	55.62	Buy	—	Buy	Buy
2021/9/30 5:30	6433.39	64.4	Buy	Buy	Sell	Sell
2021/9/30 6:00	6576.79	45.76	Sell	—	Buy	Buy
2021/9/30 6:30	6924.7	57.46	Sell	Buy	Sell	Sell
2021/9/30 7:00	6940.62	45.34	Buy	Buy	Buy	Buy
2021/9/30 7:30	6874.65	46.3	Buy	Sell	—	Buy
2021/9/30 8:00	6878.26	47.81	Buy	Buy	—	Buy
2021/9/30 8:30	6868.02	53.26	Sell	Buy	—	Sell
2021/9/30 9:00	6831.05	40.38	Buy	Buy	Buy	Buy
2021/9/30 9:30	6612.46	67.62	Sell	Buy	Sell	Sell
2021/9/30 10:00	6448.59	46.28	Buy	Buy	—	Buy
2021/9/30 10:30	6490.14	52.98	Sell	Buy	Buy	Sell
2021/9/30 11:00	6701.36	110.5	Sell	Sell	—	Sell
2021/9/30 11:30	6708.46	41.29	Sell	—	Buy	Sell
2021/9/30 12:00	6745.54	63.16	Sell	Sell	Sell	Sell
2021/9/30 12:30	6709.54	87.47	Sell	Sell	Sell	—
2021/9/30 13:00	6590.01	58.11	Buy	Sell	Sell	Sell
2021/9/30 13:30	6505.91	83.35	Buy	Sell	Sell	Sell
2021/9/30 14:00	6448.7	48.21	Sell	Sell	Buy	Buy
2021/9/30 14:30	6398.21	46.88	Sell	—	Sell	Sell
2021/9/30 15:00	6556.54	33.86	Sell	Sell	—	Buy
2021/9/30 15:30	6984.06	52.82	Sell	Sell	Sell	Buy
2021/9/30 16:00	7262.13	51.97	Sell	Sell	—	Buy
2021/9/30 16:30	7348.21	49.52	Sell	Sell	—	Buy
2021/9/30 17:00	7648.81	54.35	Buy	—	Buy	Buy
2021/9/30 17:30	7961.31	59.56	Sell	Buy	Sell	Buy
2021/9/30 18:00	8366.82	69.17	Buy	—	Sell	Sell
2021/9/30 18:30	8698.06	91.75	—	Sell	Sell	Sell
2021/9/30 19:00	8764.36	99.62	Buy	—	Sell	Sell
2021/9/30 19:30	8508.06	67.86	Sell	Sell	Sell	Sell
2021/9/30 20:00	8213.02	61.15	Sell	Sell	Sell	Sell
2021/9/30 20:30	8043.99	58.45	Buy	Sell	Sell	Buy
2021/9/30 21:00	7904.73	61.94	Buy	Sell	—	Sell
2021/9/30 21:30	7661.73	50.23	Sell	Sell	—	—
2021/9/30 22:00	7441.46	47.9	Buy	—	—	Buy
2021/9/30 22:30	7486.36	52.17	Buy	Sell	—	Buy
2021/9/30 23:00	7416.77	53.94	Sell	Sell	—	Buy
2021/9/30 23:30	7338.21	48.83	Sell	—	—	Buy
2021/10/1 0:00	7318.6	48.1	—	—	—	Buy
Total_Profit			71.3	87.03	223.32	380.58

**Table 6:** Overall evaluation of DNN-DDPG, RNN-DDPG, LSTM-DDPG, and BiLSTM-DDPG from September 29, 2021, 18:00, to October 1, 2021, 0:00, in AEMO.

Time	September 29, 2021, 18:00, to October 1, 2021, 0:00
Statistical metrics	Total_profit _($/MWh)_
DNN-DDPG	71.36
RNN-DDPG	87.03
LSTM-DDPG	223.32
BiLSTM-DDPG	380.58

## Data Availability

The data of the models and algorithms used to support the findings of this study are included within the article.

## References

[1] Du S., Wen B., Jiang C. (2004). *Power Market*.

[2] shahidehpour M. (2005). *Market Oriented Operation of Power System*.

[3] Lu Z., Han H., Shan B., Wang Y., Du S., Li J. (2017). Morphological evolution model and power forecasting prospect of future electric power systems with high proportion of renewable energy. *Power System Automation*.

[4] Kang C., Xia Q., Hu Z., Zhang B. (2004). New connotation of forecasting problem in power market. *Power System Automation*.

[5] Kang C., Yao L. (2017). Key scientific issues and theoretical research framework for power system high proportion renewable energy. *Power System Automation*.

[6] Le Ray G., Larsen E. M., Pinson P. (2016). Evaluating price-based demand response in practice-with application to the EcoGrid EU experiment. *IEEE Transactions on Smart Grid*.

[7] Ying X., Su Q., Xing W., Chiu B. C., Keech A. (2011). Impact of price responsive demand on PJM Real-time/Look-ahead markets.

[8] Nazari-Heris M., Mirzaei M. A., Mohammadi-Ivatloo B., Marzband M., Asadi S. (2020). Economic-environmental effect of power to gas technology in coupled electricity and gas systems with price-responsive shiftable loads. *Journal of Cleaner Production*.

[9] Liu G., Xu Y., Tomsovic K. (2016). Bidding strategy for microgrid in day-ahead market based on hybrid stochastic/robust optimization. *IEEE Transactions on Smart Grid*.

[10] Geng W. U., Wang H., Zeng B., Ming Z. (2019). Research on energy purchase strategy of the multi-energy service provider considering the flexible thermal load. *Electric Power Construction*.

[11] Yan Z., MARuxiang L., Zhu X., Wei Z. (2020). Multi-time scale stochastic optimal dispatch of electric vehicle charging station considering demand response. *Power System Protection and Control*.

[12] Hou H., Wang Y., Zhao B. (2022). Electric vehicle aggregator dispatching strategy under price and incentive demand response. *Power System Technology*.

[13] Zhao H., Zhang C. (2020). An online-learning-based evolutionary many-objective algorithm. *Information Sciences*.

[14] Pasha J., Dulebenets M. A., Fathollahi-Fard A. M. (2021). An integrated optimization method for tactical-level planning in liner shipping with heterogeneous ship fleet and environmental considerations. *Advanced Engineering Informatics*.

[15] Kavoosi M., Dulebenets M. A., Pasha J., Wang H., Chi H., Abioye O. F. (2019). An augmented self-adaptive parameter control in evolutionary computation: a case study for the berth scheduling problem. *Advanced Engineering Informatics*.

[16] Dulebenets M. A. (2021). An adaptive polyploid memetic algorithm for scheduling trucks at a cross-docking terminal. *Information Sciences*.

[17] Rabbani M., Oladzad-Abbasabady N., Akbarian-Saravi N. (2022). Ambulance routing in disaster response considering variable patient condition: NSGA-II and MOPSO algorithms. *Journal of Industrial and Management Optimization*.

[18] Naji S., Keivani A., Shamshirband S. (2016). Estimating building energy consumption using extreme learning machine method. *Energy*.

[19] Hu W., Zheng L., Min Y. (2017). Research on power systemtransient stability assessment based on deep learning of big data technique. *Power System Technology*.

[20] Chen Z., Liu J., Chen L. (2020). Ultra short-term power loadforecasting based on combined LSTM-XGBoost model. *Power System Technology*.

[21] Fan S., Li L., Wang S. (2020). Application analysisand exploration of artificial intelligence technology in power grid dispatch and control. *Power System Technology*.

[22] Li M., Tao H., Xu H. (2020). The technicalframework and application prospect of artificial intelligence application in the field of power grid dispatching and control. *Power System Technology*.

[23] Sun Y., Liu D., Li B. (2019). Application of deep reinforcementlearning in demand response. *Automation of Electric Power Systems*.

[24] Lu J., Cardell J. Implementing a grid state indicator for responsive retail demand.

[25] Lu Y., Liang Y., Ding Z., Wu Q., Ding T., Lee W.-J. (2022). Deep reinforcement learning based charging pricing for autonomous mobility-on-demand system. *IEEE Transactions on Smart Grid*.

[26] Amin S., Ghasemi A., Jones K. R., Bardas A. G., Hashemi M., Ahmadi R. Demand responsive dynamic pricing framework for prosumer dominated microgrids using multiagent reinforcement learning.

[27] Sang J., Sun H., Kou L. (2022). Deep reinforcement learning microgrid optimization strategy considering priority flexible demand side. *Sensors*.

[28] Liu D., Wang W., Wang L., Jia H., Shi M. (2021). Dynamic pricing strategy of electric vehicle aggregators based on DDPG reinforcement learning algorithm. *IEEE Access*.

[29] Pan Y., Wang W., Li Y., Zhang F., Sun Y., Liu D. (2021). Research on cooperation between wind farm and electric vehicle aggregator based on A3C algorithm. *IEEE Access*.

[30] Feng X., Xie T., Gao C., Lin G., Liang C., Lu S. (2019). A demand side response strategy considering long-term revenue of electricity retailer in electricity spot market. *Power System Technology*.

[31] Han D., Huang W., Zheng Y. A. N. (2022). Deep reinforcement learning for virtual bidding in electricity markets. *Proceedings of the CSEE*.

[32] Yan G., Kan T., Yang Y., Zhang W. (2020). Demand response optimal scheduling for distributed electric heating based on deep reinforcement learning. *Power System Technology*.

[33] Ibrahim M., Alsheikh A., Elhafiz R. (2022). Resiliency assessment of power systems using deep reinforcement learning. *Computational Intelligence and Neuroscience*.

[34] Liu J., Feng L. (2021). Diversity evolutionary policy deep reinforcement learning. *Computational Intelligence and Neuroscience*.

[35] Gers F. A., Schmidhuber J., Cummins F. (2000). Learning to forget: continual prediction with LSTM. *Neural Computation*.

[36] Gers F., Schmidhuber J. Recurrent nets that time and count.

[37] Quoc V., Jaitly N., Hinton G. E. (2015). A simple way to initialize recurrent networks of rectified linear units.

[38] Degris T., White M., Richard S. (2012). Off-policy actor-critic.

[39] Wang Z., Bapst V., Heess N. (2017). Sample efficient actor-critic with experience replay.

[40] Silver D., Guy L., Heess N. Deterministic policy gradient algorithms.

[41] Lillicrap T. P., Hunt J. J., Alexander P. (2019). Continuous control with deep reinforcement learning.

[42] https://www.iso-ne.com/.

[43] https://www.aemo.com.au/.

